# TIPE2 Suppresses Malignancy of Pancreatic Cancer Through Inhibiting TGFβ1 Mediated Signaling Pathway

**DOI:** 10.3389/fonc.2021.680985

**Published:** 2021-06-23

**Authors:** Fang Feng, Chunliang Liu, Huahui Bian, Wei Cai, Ying Zhou, Li Zhou, Zhixiang Zhuang

**Affiliations:** ^1^ Department of Oncology, The Second Affiliated Hospital of Soochow University, Medical College, Soochow University, Suzhou, China; ^2^ Department of Oncology, Suzhou Ninth People’s Hospital, Soochow University, Suzhou, China; ^3^ Jiangsu Institute of Hematology, The First Affiliated Hospital of Soochow University, Suzhou, China

**Keywords:** TIPE2, pancreatic cancer, malignancy, TGFβ1, tumor microenvironment

## Abstract

Pancreatic cancer is one of the major reasons of cancer-associated deaths due to poor diagnosis, high metastasis and drug resistance. Therefore, it is important to understand the cellular and molecular mechanisms of pancreatic cancer to identify new targets for the treatment. TIPE2 is an essential regulator of tumor apoptosis, inflammation and immune homeostasis. However, the role of TIPE2 is still not fully understood in pancreatic cancer. In this study, we found the expression of TIPE2 was decreased in pancreatic cancer tissues compare to paracancerous tissues, which was negatively correlated with tumor size in patients. Overexpression of TIPE2 significantly decreased cell proliferation, metastasis and increased apoptotic events in pancreatic cancer cell lines. Moreover, the results obtained from real time PCR and western blot revealed that TIPE2 was also involved in inhibiting *MMPs* and *N-Cadherin* expression while increasing Bax expression in pancreatic cancer cells. Similarly, TIPE2 could inhibit tumor growth *in vivo*, decrease the expression of Ki-67 and N-Cadherin, and increase the expression of Bax by IHC analysis in tumor tissues isolated from tumor-bearing mice. Mechanistic studies exhibited that TIPE2 might suppress pancreatic cancer development through inhibiting PI3K/AKT and Raf/MEK/ERK signaling pathways triggered by TGFβ1. Moreover, the tumor-infiltrating lymphocytes from tumor-bearing mice were analyzed by flow cytometry, and showed that TIPE2 could promote T cell activation to exert an anti-tumor effect possibly through activation of DCs in a TGFβ1 dependent manner. In general, we described the multiple regulatory mechanisms of TIPE2 in pancreatic tumorigenesis and tumor microenvironment, which suggested TIPE2 may act as a potential therapeutic target in pancreatic cancer.

## Introduction

Pancreatic cancer is one of the major causes of cancer-related mortality with a mournful 5-year survival rate of 9% (1). By the next decade, it is estimated that pancreatic cancer will rise to the second leading cause of cancer related death bypassing breast and colon cancer (2). About 90% of pancreatic cancer is pancreatic ductal adenocarcinoma, the treatment of pancreatic cancer is mainly based on comprehensive surgical intervention. However, the surgical success rate is low and the postoperative recurrence rate is high. For many years, the current standard of care for advanced pancreatic cancer is gemcitabine-based regimens (3), with an effective rate lower than 10%, and its long-term use usually leads to drug resistance (4). Even the immune checkpoint blockades, including programmed death-1/programmed death-ligand 1 (PD-1/PD-L1), and cytotoxic T lymphocyte associated antigen-4 (CTLA-4), which have been demonstrated robust results in melanoma, lung cancer, renal cell carcinoma, urothelial cancer, head and neck cancer and other malignancies (5), but most phase I and II clinical trials have failed to show any clinical efficacy in “cold tumor” pancreatic cancer (6).

As we know, pancreatic cancer patients are usually diagnosed after metastasis and/or significant local invasion have occurred, which dramatically decrease the survival rate (7). Moreover, the immunosuppressive tumor microenvironment in pancreatic cancer is another important factor, which can promote oncogenic progression (8). Therefore, it is necessary to know the mechanisms such as regulation of apoptotic signal transduction, metastasis and tumor microenvironment to identify new promising targets that may help to enable immune mediated control of this disease.

TIPE2 (tumor necrosis factor-α induced protein 8-like 2, or TNFAIP8L2), is a member of the TIPE family that is preferentially expressed in lymphoid and inflamed tissues. TIPE2 is an essential regulator of apoptosis, inflammation and immune homeostasis (9, 10). Overexpression of TIPE2 in tumor cells induced cell apoptosis and significantly inhibited Ras-induced tumorigenesis through binding to Ras-interacting domain of RGL and inhibiting the activation of Ral (9). In addition, TIPE2 inhibited invasiveness and tumor development *via* reducing MMP9 expression by targeting Rac1 in hepatocellular carcinoma (HCC) and gastrointestinal stromal tumor (11, 12). TIPE2 also inhibited HCC metastasis through suppressing Erk1/2 and NF-κB activation (13). Furthermore, TIPE2 suppressed the epithelial-mesenchymal transition (EMT) and metastasis of tumor cells *via* suppressing PI3K/Akt and Wnt/β-catenin signaling pathways in some tumor types, including glioma, gastric cancer and breast cancer (14–16). Moreover, TIPE2 could bind to β-catenin directly to decrease the occurrence of EMT and suppress metastasis of endometrial cells (17). As an important regulator of tumor microenvironment, TIPE2 was found to significantly inhibit oncogenic progression through activating T and NK cells, and inhibiting FoxP3^+^ Treg cells in tumor microenvironment (18, 19).

In this manner, TIPE2 acts as an important regulator in tumor development and tumor microenvironment during oncogenic progression. However, whether TIPE2 is involved in pancreatic cancer is still not fully understood. In this study, we describe the multiple regulatory mechanisms in pancreatic cancer development and tumor microenvironment.

## Materials And Methods

### Experimental Animals

Specific pathogen-free female nude mice (6–8 weeks) and wild type C57BL/6J (6–8 weeks) mice were purchased from Vital River Laboratory Animal Center (Beijing, China). All mice were maintained in specific pathogen-free facilities in accordance with the National Animal Care and Use Committee. The animal studies were approved by the Institutional Laboratory Animal Care and Use Committee of Soochow University.

### Immunohistochemistry

Pancreatic cancer and adjacent tissue microarray (70 cases, Outdo Biotech, Shanghai) was stained with rabbit anti-TIPE2 antibody (ProteinTech, Chicago, IL) and HRP-conjugated anti-rabbit IgG secondary antibody (Cell Signaling Technology, Danvers, MA). The immunohistochemical staining results were scored by considering the intensity of staining and the percentage of positive cells. The intensity of staining was scored as follows: 0, negative; 1, weak; 2, moderate; 3, strong. The percentage of positive stained cells was defined as follows: 0, <5%; 1, 6–25%; 2, 26–50%; 3, 51-75%; 4, 76–100%. The intensity of staining multiplies the percentage of positive cells to produce a final score of TIPE2 expression as follows: 0, total score = 0; 1+, total score = 1–4; 2+, total score =5–8; 3+, total score = 9–12.

Tumor tissues isolated from mice were fixed in 10% neutral buffered formalin and embedded in paraffin. Slides were stained with primary antibody, including anti-TIPE2 (ProteinTech), anti-Ki67 (Abcam, Cambridge, MA), anti-TGFβ1 (ProteinTech), anti-Bax (Cell Signaling Technology) and anti-N-cadherin (Cell Signaling Technology), and then stained with HRP anti-rabbit IgG secondary antibody (Cell Signaling Technology).

### Cell Culture

AsPC-1, BxPC-3 and 293T cell lines were purchased from the Cell Bank of Chinese Scientific Academy (Shanghai, China), Panc02 cell line was purchased from the National Experimental Cell Resource Sharing platform (Beijing, China). No mycoplasma contamination was detected in these cell lines by PCR analysis. AsPC-1 and BxPC-3 cells were cultured in 10% FBS RPMI-1640. Panc02 and 293T cells were cultured in 10% FBS DMEM.

In AsPC-1 cells, the neutralizing anti-TGFβ1 monoclonal antibody (1 μg/ml, ProteinTech) or 5 ng/ml human recombinant TGFβ1 (ProteinTech) was added for 48 h incubation, respectively.

### Overexpression of TIPE2 in Pancreatic Cancer Cell Lines

The full coding sequences of human and murine TIPE2 were amplified and subcloned into lentiviral vector pCAG. The lentivirus encoding TIPE2 was produced by co-transfecting 293T cells with pCAG and lentivirus packaging plasmids. Empty pCAG vector was used as vector control. To overexpress TIPE2 in pancreatic cancer cell lines (AsPC-1, BxPC-3 and Panc02), all cell lines were infected with TIPE2-expressing lentivirus and vector control lentivirus. The pancreatic cancer cell lines stably expressing TIPE2 or vector control were constructed by sorting GFP positive cells using BD FACS flow cytometer (San Jose, CA), and the expression of TIPE2 was confirmed by western blot analysis.

### Cell Proliferation and Cell Apoptosis Assay

For cell proliferation assay, the cell proliferation was examined using cell counting kit-8 (CCK-8, Dojindo, Kumamoto, Japan) as described by the manufacturer’s protocol. Cells were seeded in 96-well plate at a destiny of 3,000 cells/well (AsPC-1 and BxPC-3 cell lines) and 1,000 cells/well (Panc02 cell line), and then cultured for 0, 24, 48 and 72 h. At the indicated time point, 10 μl of CCK-8 solution was added to each well and incubated for additional 3 h. The optical density (OD) value was measured at 450 nm on a microplate reader (BioTek, Winooski, VT).

For cell apoptosis assay, cells were seeded in 6-well plate at a destiny of 2 × 10^5^ cells/well (AsPC-1 and BxPC-3 cell lines) and 1 × 10^5^ cells/well (Panc02 cell line). The cells were collected 48 h after incubation, then stained with Annexin V and 7-AAD (BD Biosciences, San Diego, CA), and were analyzed using flow cytometry.

### Migration and Invasion Assay

The highly metastatic pancreatic cancer cell line, AsPC-1, was used for cell migration and invasion analysis using transwell chambers (Corning, New York, NY) with 8-μm pore size membrane inserts in 24-well plate. And the membrane inserts were coated with matrigel (BD Biosciences) for invasion assay. Some 150 μl cells (1× 10^5^/ml) were resuspended in serum-free medium and seeded onto the upper chambers with or without matrigel. The bottom chambers were filled with 600 μl RPMI-1640 medium supplemented with 10% FBS. After 16 h in migration assay and 24 h in invasion assay, the cells passed through the membrane were fixed with methanol and stained with 0.1% crystal violet. The stained cells were photographed and counted under the microscope.

### Quantitative Real Time PCR Analysis

Total RNA was extracted from cells and was reverse-transcribed into cDNA by reverse transcription kit (TaKaRa, Otsu, Japan). The relative gene expression was detected by real-time PCR performed using SYBR Green PCR master mixes (Thermo Fisher, Waltham, MA). GAPDH was used as the internal control. The primers used for these analyses are listed in **Supplementary Table 1**.

### Xenograft Tumor Model

Human AsPC-1/vector and AsPC-1/TIPE2 cells, BxPC-3/vector and BxPC-3/TIPE2 cells (1 × 10^6^ cells in 100 μl PBS per mouse) were injected subcutaneously into the right hind legs of BALB/c nude mice (6–8 weeks). After ten days, the mice were monitored weekly for tumor size using a caliper. The tumor volume was calculated according to the standard formula: volume = 0.5 × length × width^2^. The mice were sacrificed when tumor volume reached approximate 400 mm^3^, then the tumor tissues were isolated and photographed.

### Western Blot Analysis

Total protein was extracted from cell lines lysed with RIPA buffer (Beyotime, Shanghai, China) in the presence of a protease inhibitor and phosphatase inhibitor (Selleck, Shanghai, China). The concentration of protein was determined by BCA kit (Beyotime). Protein was resolved by SDS-PAGE and then transferred to polyvinylidene difluoride (PVDF) membrane (Millipore, Burlington, MA). The membranes were blocked with 5% non-fat milk in TBST for 2 h at room temperature and then incubated with primary antibodies overnight at 4°C. The primary antibodies include rabbit anti-TIPE2 (ProteinTech), anti-pERK, anti-ERK, anti-pAKT, anti-AKT, anti-Bax (Cell Signaling Technology), anti-GAPDH (ProteinTech), anti-pTGFBR1 and anti-TGFBR1 (Affinity, Cincinnati, OH). After washing, membranes were incubated with HRP-conjugated secondary antibody (goat anti-rabbit IgG or goat anti-mouse IgG, Cell Signaling Technology) for 1 h at room temperature. After washing, the protein bands were visualized with enhanced chemiluminescence (ECL) detection kit (Beyotime).

### ELISA Analysis

AsPC-1/vector, AsPC-1/TIPE2 cells, Panc02/vector and Panc02/TIPE2 cells were seeded in 6-well plate at a destiny of 2 × 10^5^ and 1 × 10^5^. The supernatants were collected after 48 h of incubation and the protein level of TGFβ1 was analyzed using human TGFβ1 ELISA kit (Proteintech) and mouse TGFβ1 ELISA kit (Biosensis, Thebarton, South Australia).

### Subcutaneous Tumor Model

Murine Panc02/vector and Panc02/TIPE2 cells (1 × 10^6^ cells in 100 μl PBS per mouse) were injected subcutaneously into the right hind legs of female C57BL/6J mice. After one week, the mice were monitored weekly for tumor size. The tumor volume was calculated by the formula: volume = 0.5 × length × width^2^. The mice were sacrificed when tumor volume reached approximate 400 mm^3^, then the tumor tissues were isolated, weighted and photographed. Then tumor-infiltrating lymphocytes were isolated for flow cytometry analysis.

### Flow Cytometry Analysis

For analysis of tumor-infiltrating lymphocytes (TILs), tumor-infiltrating cells were isolated from tumors. The antibodies used for FACS staining including anti-mouse CD3ϵ, CD4, CD8α, CD69, CD62L, CD44, CD11c, CD80 and PD-L1 were purchased from Biolegend (San Diego, CA). Flow cytometry analysis was performed using a Novocyte flow cytometer (Agilent, Carpinteria, CA).

### Dendritic Cell (DC) Culture *In Vitro*

Bone marrow cells isolated from C57BL/6 mice were culture in 6-well plates (1 × 10^6^ cells/ml, 4 ml/well) in the presence of 20 ng/ml IL-4 and 40 ng/ml GM-CSF. On day 2, change the cell culture medium fully and softly with fresh RPMI-1640 medium containing IL-4 and GM-CSF. On days 4 and 6, replace one half of culture medium with fresh complete RPMI-1640 medium. On day 7, the DCs were harvested and co-cultured with Panc02/vector or Panc02/TIPE2 cells. After 24 h, the cells were harvested and analyzed with flow cytometry.

### Statistical Analysis

Results were presented as mean ± SD. Statistical test was performed with student’s t test (unpaired, two-tailed) using Graphpad prism 5.0 software (Graphpad, San Diego, CA). For all analysis, P <0.05 was considered to indicate a statistically significant difference.

## Results

### TIPE2 Expression Was Decreased in Human Pancreatic Carcinoma

**Figure 1:**
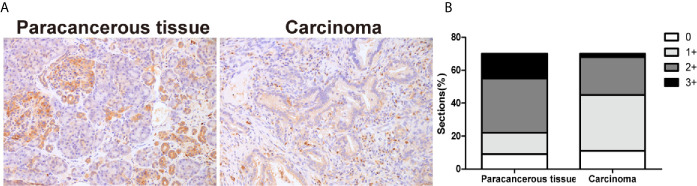
The expression of TIPE2 in human pancreatic carcinoma tissues. **(A)** TIPE2 expression was examined in pancreatic carcinoma and paracancerous tissues with rabbit anti-TIPE2 antibody using immunohistochemistry. The positive cells were stained brown. Original magnification: 200×. **(B)** All tissues were scored by considering the intensity of staining and the percentage of positive cells.

The expression of TIPE2 was analyzed in pancreatic carcinoma and paracancerous tissues using immunohistochemistry. The proportion and the staining intensity of TIPE2-positive cells were obviously lower in carcinoma than in paracancerous tissue (**Figures 1A, B** **)**. To further investigate the clinical significance of TIPE2 expression in pancreatic cancer, we analyzed the relationship between TIPE2 expression and clinico-pathological features (**Table 1**). TIPE2 expression was negatively correlated with tumor size (*P = 0.014*), while there was no significant correlation between TIPE2 expression and age, gender, TNM stage or lymph node metastasis. These data suggested that TIPE2 might play an important role in the progression of pancreatic cancer.

**Table 1:** Relationship between TIPE2 expression and clinico-pathological features in pancreatic cancer.

Clinico-pathological features	Number	TIPE2		*P*
		Low	High	
**Age**				
<60	27	18	9	0.742
≥60	43	27	16	
**Gender**				
Male	46	29	17	0.764
Female	24	16	8	
**Tumor Size**				
<5cm	49	27	22	0.014
≥5cm	21	18	3	
**TNM Stage**				
I + II	48	30	18	0.645
III + IV	22	15	7	
**Lymph node metastasis**				
Positive	23	16	7	0.519
Negative	47	29	18	

### Overexpression of TIPE2 Inhibited the Proliferation of Pancreatic Cancer Cells and Increased Cell Apoptosis

**Figure 2:**
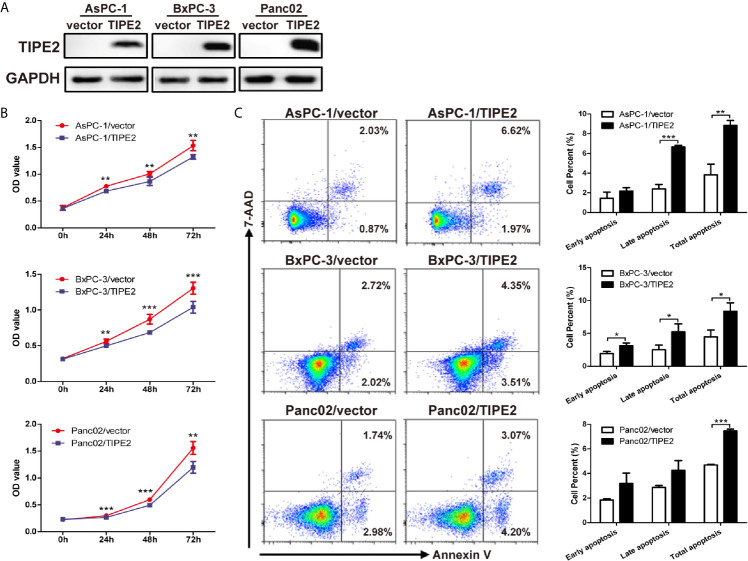
Overexpression of TIPE2 inhibited cell proliferation and increased apoptosis of pancreatic cancer cells. **(A)** TIPE2-overexpressing or vector control cells were established by infecting with lentivirus and sorting with flow cytometry. The expression of TIPE2 was confirmed by western blot analysis. **(B)** Cell viability of TIPE2-overexpressing or vector control cells was examined using CCK-8. **(C)** Cells were stained with Annexin V and 7-AAD and analyzed by flow cytometry to detect the cell apoptosis (Annexin V^+^/7-AAD^−^, early apoptosis: Annexin V^+^/7-AAD^+^, late apoptosis). Data shown were representative of three independent experiments. Values are presented as means ± SD. *p < 0.05, **p < 0.01, ***p < 0.001.

To evaluate the role of TIPE2 in pancreatic cancer, we overexpressed TIPE2 in pancreatic cancer cell lines with lentivirus, including human cell lines AsPC-1 and BxPC-3, and mouse cell line Panc02, which have low level of TIPE2 expression. And we demonstrated that TIPE2 was successfully overexpressed in these cell lines compared with vector controls by western blot analysis (**Figure 2A**). To assess the function of TIPE2 in tumor cell biological activity *in vitro*, the cell proliferation and cell apoptosis assays were performed. TIPE2 markedly inhibited cell proliferation of pancreatic cancer cells using CCK-8 assay (**Figure 2B**). TIPE2 expression also significantly increased cell apoptosis of the pancreatic cancer cells (**Figure 2C**).

### TIPE2 Suppressed the Metastasis of Pancreatic Cancer Cells *In Vitro*

**Figure 3:**
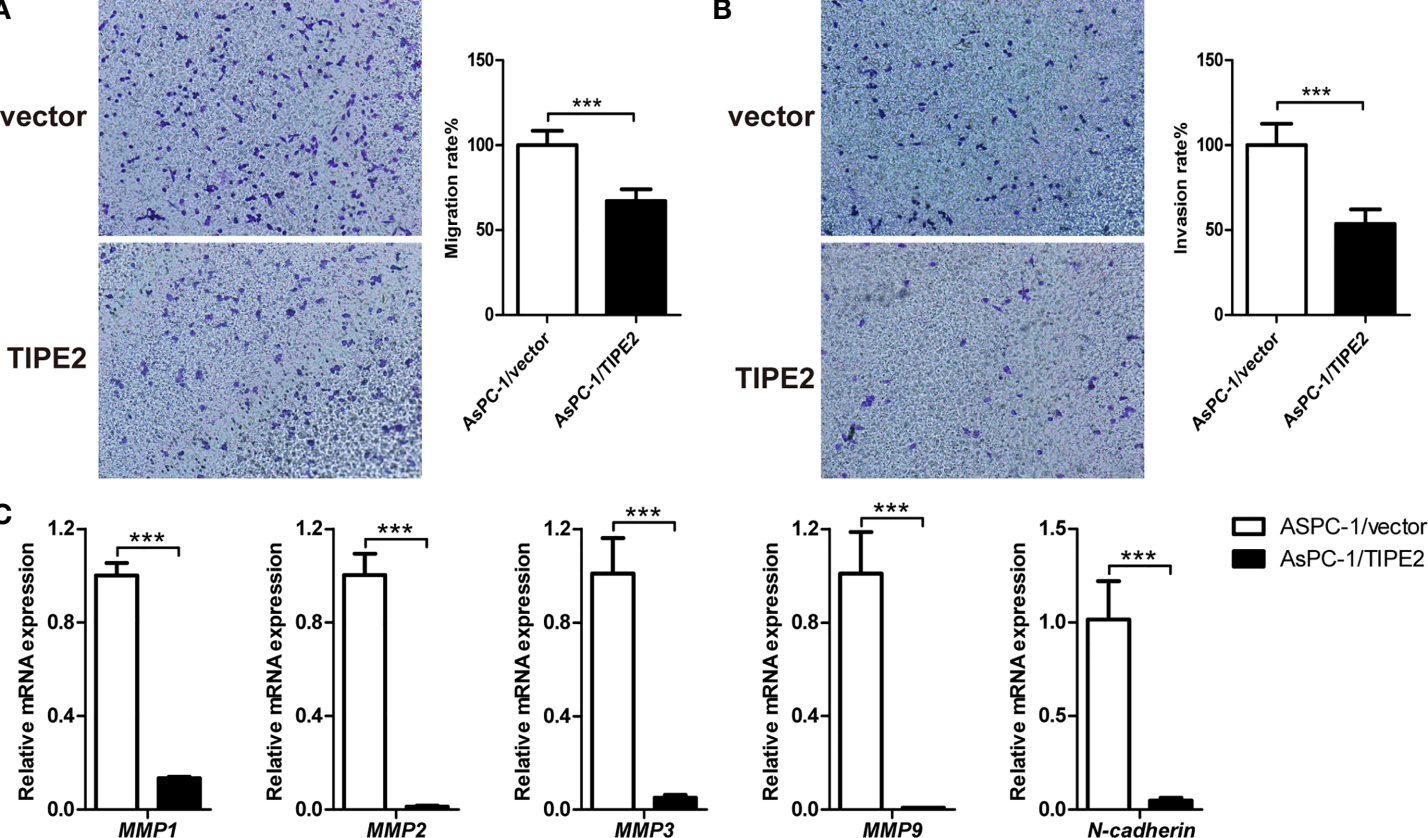
TIPE2 suppressed the metastasis of pancreatic cancer cells in vitro. **(A, B)** Cell migration ability was determined with transwell assay, while cell invasion ability was determined with matrigel-coated transwell assay. The cells on the bottom side of the chamber were fixed, stained and counted. **(C)** Real-time qPCR analysis of *MMP1*, *MMP2*, *MMP3*, *MMP9* and *N-cadherin* expression. The data shown are the representative of three independent experiments. Values are presented as means ± SD. ***p < 0.001.

To investigate the function of TIPE2 in the migration and invasion of pancreatic cancer cells *in vitro*, we performed transwell assays using the highly metastatic pancreatic cancer cell line AsPC-1. TIPE2 overexpression could reduce the number of migrated and invaded cells (**Figures 3A, B** **)**. The results showed that TIPE2 suppressed both the migration and invasion abilities of pancreatic cancer cells. To further explore the mechanism of TIPE2 in the metastasis of pancreatic cancer cells, we detected the expression of EMT markers, *MMPs* and *N-cadherin*. Overexpression of TIPE2 significantly reduced the expression of *MMP1*, *MMP2*, *MMP3*, *MMP9* and *N-cadherin* (**Figure 3C**). Therefore, TIPE2 might suppress the metastasis of pancreatic cancer cells through inhibiting the EMT process.

### TIPE2 Suppressed Tumorigenesis of Pancreatic Cancer in Xenograft Tumor Models

**Figure 4:**
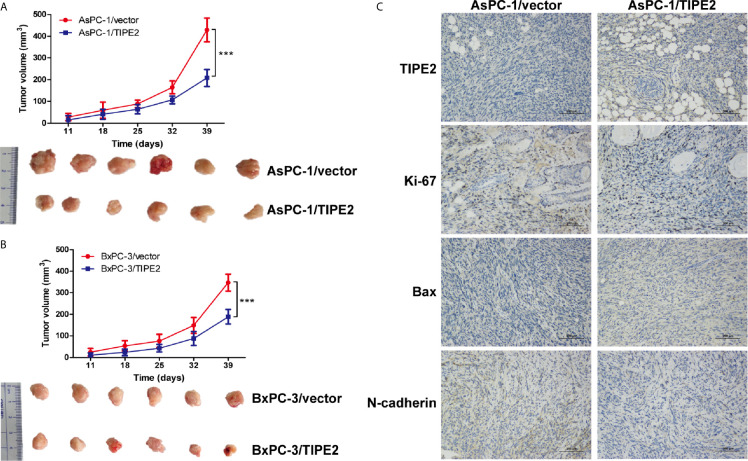
TIPE2 suppressed the growth of pancreatic cancer in vivo. **(A, B)** The tumor volume was measured weekly ten days after tumor inoculation. Subcutaneous tumor growth curve was calculated. The tumors were isolated from nude mice and photographed 40 days after tumor cells inoculation. **(C)** TIPE2, Ki-67, Bax and N-cadherin expressions were examined in tumor tissues using immunohistochemistry. The data shown are the representative of three independent experiments. Values are presented as means ± SD.***p < 0.001.

To explore the role of TIPE2 in pancreatic tumorigenesis *in vivo*, we established the subcutaneous xenograft tumor model using nude mice. The tumor volume was measured weekly from 10 days after tumor cell inoculation. TIPE2 significantly reduced the tumor volume compared with vector group during the progression of pancreatic cancer (**Figures 4A, B** **)**. Then the tumors were isolated from mice which were sacrificed around 40 days after tumor cells injection. The tumor size of TIPE2 group was significantly smaller than that of vector group (**Figures 4A, B** **)**. And we also confirmed TIPE2 was overexpressed in the pancreatic tumor tissues isolated from mice through immunohistochemical staining (**Figure 4C**). Moreover, TIPE2 overexpression could decrease the expression of Ki-67 and N-cadherin, and increase the expression of Bax (**Figure 4C**). These results showed that TIPE2 could affect tumor cell proliferation, apoptosis and EMT process, which demonstrated that TIPE2 could suppress the progression of pancreatic cancer *in vivo*.

### TIPE2 Inhibited PI3K/AKT and Raf/MEK/ERK Signaling Pathways Triggered by TGFβ1

**Figure 5:**
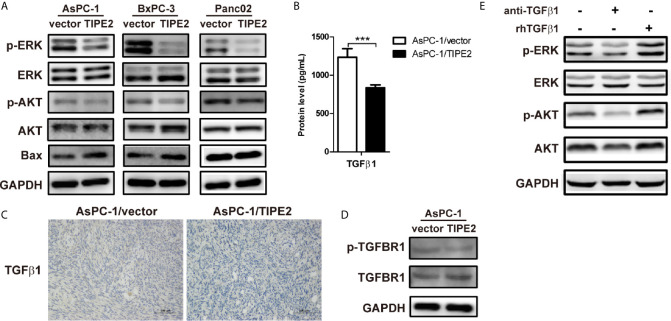
TIPE2 inhibited PI3K/AKT and Raf/MEK/ERK signaling pathways triggered by TGFβ1. **(A)** The total proteins extracted from cells were analyzed for the expression of AKT, ERK and Bax using western blot. The antibodies used were anti-pAKT, anti-AKT, anti-pERK, anti-ERK, anti-Bax and anti-GAPDH. **(B)** ELISA analysis of TGFβ1 secretion from AsPC-1/vector and AsPC-1/TIPE2 cells. **(C)** Immunohistochemistry analysis of the TGFβ1 expression in AsPC-1/vector and AsPC-1/TIPE2 tumor tissues. **(D)** Western blot analysis of the expression of p-TGFBR1 and total TGFBR1 in AsPC-1/vector and AsPC-1/TIPE2 cells. **(E)** AsPC-1 cells were seeded in 6-well plate and added with or without anti-TGFβ1 antibody or rhTGFβ1 protein. After 48 h incubation, the total proteins extracted from the cultured AsPC-1 cells were analyzed for the expression of AKT and ERK using western blot. The antibodies used were anti-pAKT, anti-AKT, anti-pERK, anti-ERK and anti-GAPDH. Data shown were representative of three independent experiments. Values are presented as means ± SD. ***p < 0.001.

Furthermore, we tried to peek into the mechanism of TIPE2 that mediates the function of pancreatic cancer cells. We detected the expression AKT, ERK and Bax which were involved in tumor proliferation, apoptosis, metastasis and EMT process. TIPE2 overexpression reduced the phosphorylation of ERK and AKT in all three cell lines (**Figure 5A**), and upregulated Bax expression in AsPC-1 and BxPC-3 cell lines (**Figure 5A**). As we know, TGFβ1 plays a key role in triggering PI3K/AKT and Raf/MEK/ERK signaling pathways (20, 21), and we found that TIPE2 overexpression could reduce TGFβ1 secretion from AsPC-1 cells (**Figure 5B**), and similarly decreased the protein level of TGFβ1 in tumor tissues of tumor-bearing mice (**Figure 5C**). Moreover, the phosphorylation of TGFBR1 level was also reduced in TIPE2 overexpression cells (**Figure 5D**). Furthermore, we demonstrated that phosphorylation of ERK and AKT was decreased after blocking TGFβ1 with anti-TGFβ1 antibody and increased after incubated with rhTGFβ1 protein in AsPC-1 cells (**Figure 5E**). These results suggested TIPE2 might affect pancreatic cancer cells *via* inhibiting PI3K/AKT and Raf/MEK/ERK signaling pathways triggered by TGFβ1.

### TIPE2 Suppressed the Growth of Pancreatic Cancer Through Inhibiting TGFβ1 Expression in Subcutaneous Tumor Model

**Figure 6:**
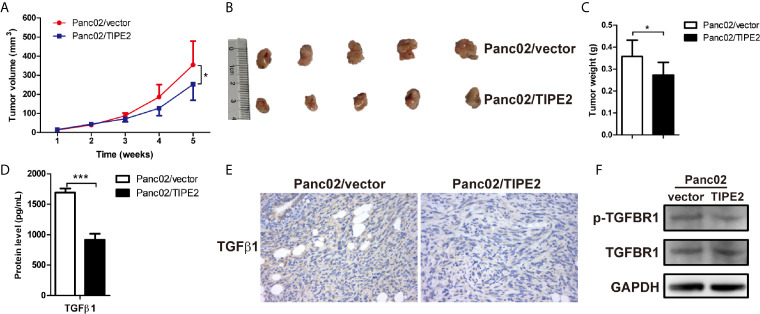
TIPE2 suppressed the growth of pancreatic cancer through inhibiting TGFβ1 expression in subcutaneous tumor model. **(A)** The tumor volume was measured weekly one week after tumor inoculation and the tumor growth curve was calculated. **(B, C)** The tumors were isolated, weighted and photographed five weeks after tumor cells inoculation. **(D)** ELISA analysis of TGFβ1 secretion from Panc02/vector and Panc02/TIPE2 cells. **(E)** Immunohistochemistry analysis of the TGFβ1 expression in Panc02/vector and Panc02/TIPE2 tumor tissues. **(F)** Western blot analysis of the expression of p-TGFBR1 and total TGFBR1 in Panc02/vector and Panc02/TIPE2 cells. The data shown are the representative of three experiments. Values are presented as means ± SD. *p < 0.05, ***p < 0.001.

TIPE2 can regulate tumorigenesis not only directly from the inside of tumor cells, but also indirectly *via* immune cells. To explore the immune function of TIPE2 in the development of pancreatic cancer *in vivo*, we injected Panc02/vector and Panc02/TIPE2 cells into C57BL/6 mice to establish the subcutaneous tumor model. The sizes of tumors were measured weekly one week after tumor cell inoculation. And the results showed that the tumor sizes of TIPE2 group were significantly smaller than vector group during the development of pancreatic cancer (**Figure 6A**). The mice were sacrificed five weeks after injection of tumor cells and the tumors were isolated, weighted and photographed. The tumor size and weight of TIPE2 group were obviously less than vector group (**Figures 6B, C** **)**. The protein levels of TGFβ1 secreted from cells and expressed in tumor tissues of tumor bearing mice were both detected by ELSIA and IHC, and the expression of TGFBR1 was detected by western blot. Similarly, TIPE2 overexpression could decrease the protein level of TGFβ1 (**Figures 6D, E** **)** and phosphorylation of TGFBR1 (**Figure 6F**) in pancreatic cancer. Therefore, TIPE2 possibly suppressed the growth of pancreatic cancer through inhibiting TGFβ1 expression.

### TIPE2 Promoted T Cell Activation to Exert Anti-Tumor Effect Through Activation of DCs in Mouse Pancreatic Cancer Model

**Figure 7:**
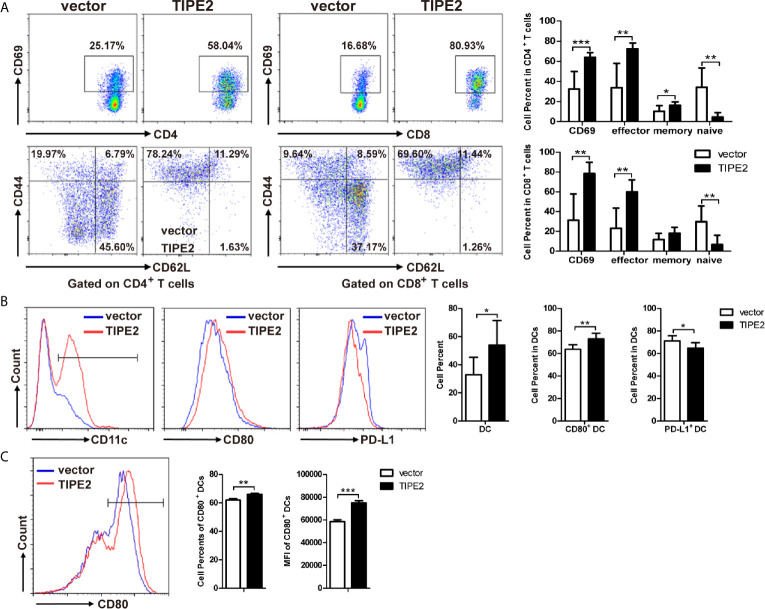
TIPE2 promoted T cell activation to inhibit the development of pancreatic cancer through activation of DCs. **(A)** Flow cytometry analysis of activated (CD69^+^) CD4^+^ T and CD8^+^ T cells, and the effector (CD62L^-^CD44^+^) of CD4^+^ T and CD8^+^ T cells in TILs. Flow cytometric plots and the summary results were shown. **(B)** Flow cytometry analysis of DCs, CD80^+^ DCs and PD-L1^+^ DCs in tumor-infiltrating lymphocytes. **(C)** Co-culture DCs and Panc02/vector or Panc02/TIPE2 cells for 24 h. Flow cytometry analysis the activation of DCs (CD80^+^ DCs). Flow cytometric plots and the summary results of percentage and mean fluorescence intensity were shown. The data shown are the representative of three experiments. Values are presented as means ± SD. *p < 0.05, **p < 0.01, ***p < 0.001.

To investigate the mechanism of the immune antitumor effect of TIPE2 in pancreatic cancer, we analyzed the tumor-infiltrating lymphocytes *via* flow cytometry. We found that TIPE2 could increase the percentage of activated (CD69^+^) and effector (CD62L^-^CD44^+^) CD4^+^ T and CD8^+^ T cells (**Figure 7A**). The percentage of DCs was also increased in TIPE2 group mice. Moreover, the percentage of activated CD80^+^ DCs was increased, and the percentage of PD-L1^+^ DCs was decreased in TIPE2 group (**Figure 7B**). How does TIPE2 affect the activation of DCs? Previous study has reported TGFβ1 could modulate the functions of DCs (22). And we have demonstrated that TIPE2 suppressed the expression of TGFβ1. Then we co-cultured the Panc02 cells with DCs *in vitro*. The result showed that TIPE2 also increased the percentage and mean fluorescence intensity of CD80^+^ DCs (**Figure 7C**). Therefore, we inferred that TIPE2 might promote T cell activation to exert anti-tumor effect through activating DCs in a TGFβ1 dependent manner.

## Discussion

TIPE2 belongs to TNFAIP8 family which consists of TIPE, TIPE1, TIPE2 and TIPE3. TIPE2 is preferentially expressed in lymphoid tissues and a variety of specific epithelial tissues such as pancreas, thymus, and kidney (23). Preceding studies have established TIPE2 reveals vital roles in inflammation and cancer. In human cancer, TIPE2 is weakly expressed in hepatocellular carcinoma (9), small cell lung cancer (24), gastric cancer (25), breast cancer (26), esophageal cancer (27) and oral tongue squamous cell carcinoma (19), which demonstrated that TIPE2 was involved in cancer progression. In our current study, the expression of TIPE2 was significantly decreased in pancreatic cancer compared to paracancerous tissues, that is consistent with other types of cancers. The expression of TIPE2 was negatively correlated with tumor size in patients. These results indicated that TIPE2 might play an important role in the development of pancreatic cancer.

To further evaluate the regulation role of TIPE2 in the malignancy of pancreatic cancer, we constructed pancreatic cancer cell lines overexpressing TIPE2 or vector control. Overexpression of TIPE2 in pancreatic cancer cells could inhibit cell proliferation, and increase cell apoptosis. Meanwhile, overexpression of TIPE2 suppressed the migration and invasion of pancreatic cancer cells *via* decreasing the expression of *MMPs* and *N-cadherin*. As indicated in other studies, both MMPs-mediated extracellular matrix degradation/remodeling and N-cadherin were involved in EMT process and metastasis of pancreatic cancer (28–31). Furthermore, TIPE2 indeed reduced the expression of Ki-67 and N-cadherin, increased the expression of Bax, and suppressed the tumor growth of pancreatic cancer in mouse xenograft tumor models.

It has been demonstrated that PI3K/AKT and Raf/MEK/ERK signaling pathways could mediate cell apoptosis, proliferation and metastasis in pancreatic cancer (28, 32–34). We demonstrated that TIPE2 could reduce the phosphorylation of ERK and AKT in pancreatic cancer cells. We also found that TIPE2 could upregulate the expression of pro-apoptotic protein Bax, while Bax was involved in the regulation of apoptosis and could influence the prognosis of pancreatic cancer patients (35). As reported, TGFβ1 is a dual character cytokine during tumorigenesis, and considered to contribute tumor progression by inducing EMT, promoting tumor immune evasion and resisting apoptosis in pancreatic cancer (36). TGFβ1 triggers Raf/MEK/ERK and PI3K/AKT signaling pathways to promote tumor malignancy in some types of cancers (20, 21). TGFBR1 is a key receptor of TGFβ1, and the phosphorylation of TGFBR1 is involved in these processes (37, 38). Indeed, we found that blocking TGFβ1 with anti-TGFβ1 antibody could reduce the phosphorylation of ERK and AKT in pancreatic cancer, while treating with TGFβ1 protein showed the opposed effect. Furthermore, TIPE2 could not only inhibit the secretion of TGFβ1, but also decrease the phosphorylation of TGFBR1 in pancreatic cancer cells. These results suggested that TIPE2 might affect pancreatic cancer *via* inhibiting PI3K/AKT and Raf/MEK/ERK signaling pathways triggered by TGFβ1.

TIPE2, as a crucial regulator in immune responses and homeostasis, involved in both innate and adaptive immune responses, can regulate tumorigenesis not only directly from the inside of tumor cells, but also indirectly *via* immune cells (39). To explore the immune-regulation role of TIPE2 in pancreatic cancer, we established the subcutaneous tumor model through injecting mouse Panc02/vector and Panc02/TIPE2 cells into mice. TGFβ1 is a main factor inducing immunosuppression in tumor microenvironment of pancreatic cancer, and inhibition of TGFβ1 strategy may provide a new opportunity for the treatment of pancreatic cancer (36). Therefore, we speculated that TIPE2 may play an anti-tumor role through inhibiting TGFβ1 in tumor microenvironment. Indeed, TIPE2 inhibited the growth of pancreatic cancer in mice subcutaneous tumor model and the expression of TGFβ1 in tumor cells was suppressed. Furthermore, we found that TIPE2 could promote T cell and DC activation to inhibit the development of pancreatic cancer. The upregulating of costimulatory molecules on DCs which interact with CD28 receptor molecules on the T cells could promote T cell activation (40, 41). TIPE2 also upregulated the expression of CD80 on DCs. Therefore, TIPE2 may activate T cells through promoting the activation of DCs. Then we found that TIPE2 could decrease the cell percentage of PD-L1^+^ DCs in tumor-infiltrating lymphocytes. According to the report that PD-L1 expression on DCs could attenuate T cell activation recently (42), we speculated that TIPE2 could activate T cells not only through activating DCs, but also through inhibiting PD-L1 expression on DCs. But why TIPE2 could activate DCs and inhibit PD-L1 expression on DCs? TGFβ1, as one of the most important factors of modulating the function of DCs in tumor microenvironment, has been proved that could induce DCs tolerance directly, or increase the expression of PD-L1 on DCs in a STAT3 dependent manner (22, 43). Additionally, we also found that TIPE2 could activate DCs *in vitro*. Therefore, TIPE2 might exert an anti-tumor effect by activation T cells through DCs in a TGFβ1 dependent manner in pancreatic cancer. However, the regulation mechanism of TGFβ1 regulated by TIPE2 is not clear, which need to be further investigated.

Taken together, our present study demonstrated that the expression of TIPE2 was reduced in human pancreatic cancer, which was negatively correlated with tumor size. Overexpression of TIPE2 significantly suppressed the proliferation, metastasis, and promoted apoptosis of pancreatic cancer possibly through inhibition of PI3K/AKT and Raf/MEK/ERK signaling pathways triggered by TGFβ1. Moreover, TIPE2 promoted T cell activation to exert an anti-tumor effect possibly through activation of DCs in a TGFβ1 dependent manner. In summary, we described the multiple regulatory mechanisms of TIPE2 in pancreatic tumorigenesis and tumor microenvironment, based on the result obtained it is suggested that TIPE2 may serve as a potential therapeutic target in pancreatic cancer.

## Data Availability Statement

The original contributions presented in the study are included in the article/**Supplementary Material**. Further inquiries can be directed to the corresponding authors.

## Ethics Statement

The animal study was reviewed and approved by the Institutional Laboratory Animal Care and Use Committee of Soochow University.

## Author Contributions

ZZ, FF and CL conceived and designed the study. FF, CL, HB, YZ and LZ performed the experiments. FF, CL and WC analysed the data. ZZ, FF and CL wrote and revised the manuscript. All authors contributed to the article and approved the submitted version.

## Funding

This work was supported by National Natural Science Foundation of China (NSFC, 81903248) and the project funding from Suzhou Ninth People’s Hospital (YK202036).

## Conflict of Interest

The authors declare that the research was conducted in the absence of any commercial or financial relationships that could be construed as a potential conflict of interest.
