# Supplementary material for: TIPE2 Suppresses Malignancy of Pancreatic Cancer Through Inhibiting TGFβ1 Mediated Signaling Pathway
Source: Front Oncol. 2021 Jun 23;11:680985. doi: 10.3389/fonc.2021.680985 (PMC8260882; doi:10.3389/fonc.2021.680985)
Supplement: Supplementary file 1 [file Table_1.docx]

**Table S1. Primer sequences used for Real time qPCR.**

| Gene | **Primer sequence** |
| --- | --- |
| Human MMP1 | Forward: 5’-GGGGCTTTGATGTACCCTAGC-3’ |
|  | Reverse: 5’-TGTCACACGCTTTTGGGGTTT-3’ |
| Human MMP2 | Forward:5’-GATACCCCTTTGACGGTAAGGA-3’ |
|  | Reverse: 5’-CCTTCTCCCAAGGTCCATAGC-3’ |
| Human MMP3 | Forward: 5’-CTGGACTCCGACACTCTGGA-3’ |
|  | Reverse: 5’-CAGGAAAGGTTCTGAAGTGACC-3’ |
| Human MMP9 | Forward: 5’-AGACCTGGGCAGATTCCAAAC-3’ |
|  | Reverse: 5’-CGGCAAGTCTTCCGAGTAGT-3’ |
| Human N-cadherin | Forward: 5’-TGCGGTACAGTGTAACTGGG-3’ |
|  | Reverse: 5’-GAAACCGGGCTATCTGCTCG-3’ |
| Human GAPDH | Forward: 5’-GGAGCGAGATCCCTCCAAAAT-3’ |
|  | Reverse: 5’-GGCTGTTGTCATACTTCTCATGG-3’ |
